# Hand sanitizers as a preventive measure in COVID-19 pandemic, its characteristics, and harmful effects: a review

**DOI:** 10.1186/s42506-021-00094-x

**Published:** 2022-02-08

**Authors:** Parixit Prajapati, Heli Desai, Chandni Chandarana

**Affiliations:** SSR College of Pharmacy, Sayli, Silvassa, UT of Dadra and Nagar Haveli 396230 India

**Keywords:** Hand sanitizer, COVID-19, Pandemic, Prevention, Innovation techniques, Harmful effects

## Abstract

**Background:**

In the global health emergency caused by COVID-19, multiple experts have mandated the use of hand sanitizers as a safety measure from COVID-19. The sale of hand sanitizers has increased many folds. Therefore, when there is such large use of hand sanitizers, it becomes extremely important to study and understand hand sanitizers in a comprehensive manner.

**Main body of the abstract:**

This article starts with the importance of sanitizers as a defence mechanism that is employed by the hand to fight against the coronavirus. This article provides information about history, types, composition, various dosage forms, and marketed formulations of hand sanitizers. The article sheds a detailed light on industrial production techniques for hand sanitizers and also outlines new innovative techniques that were employed by the industry to mass produce hand sanitizers in the wake of the pandemic. The article further dives into a comparison between hand sanitizers and soaps so as to give pros and cons of the use of soap against the use of hand sanitizers. One of the aims of the article is to study the side effects of sanitizers so as to develop a cautious approach while using hand sanitizers and therefore a comprehensive list of side effects of the use of hand sanitizers is given.

**Conclusion:**

The review article finds that hand sanitizers are extremely efficient in fight the virus but along with it, it brings along arrange of risks which are outlined in the article.

## Background

Hand sanitizer, also known as a hand antiseptic or hand rub, is a product that is applied to the hands to remove common pathogen in the hands [1]. Hand sanitizers are usually available as foam, gel, or liquid [2–4]. They are recommended for use when there is unavailability of water and soap or there are other medical concerns (e.g., it causes cracks on the skin) [5, 6]. In early 2020, WHO declared a pandemic “severe acute respiratory syndrome coronavirus 2”, better known as COVID-19. Exponential rise has been seen in the cases despite authorities setting down their best efforts. Prophylaxis is the easiest method to reduce transmission, proper hand washing and hygiene are the most effective pandemic strategies [7]. Hand sanitizers have emerged to be alternative to soap and water washing both in healthcare and public institutions. They are used to break the chain of infections, making them one of the important protocols for reducing the burden on healthcare [8].

## History of hand sanitizers

**Fig. 1:**
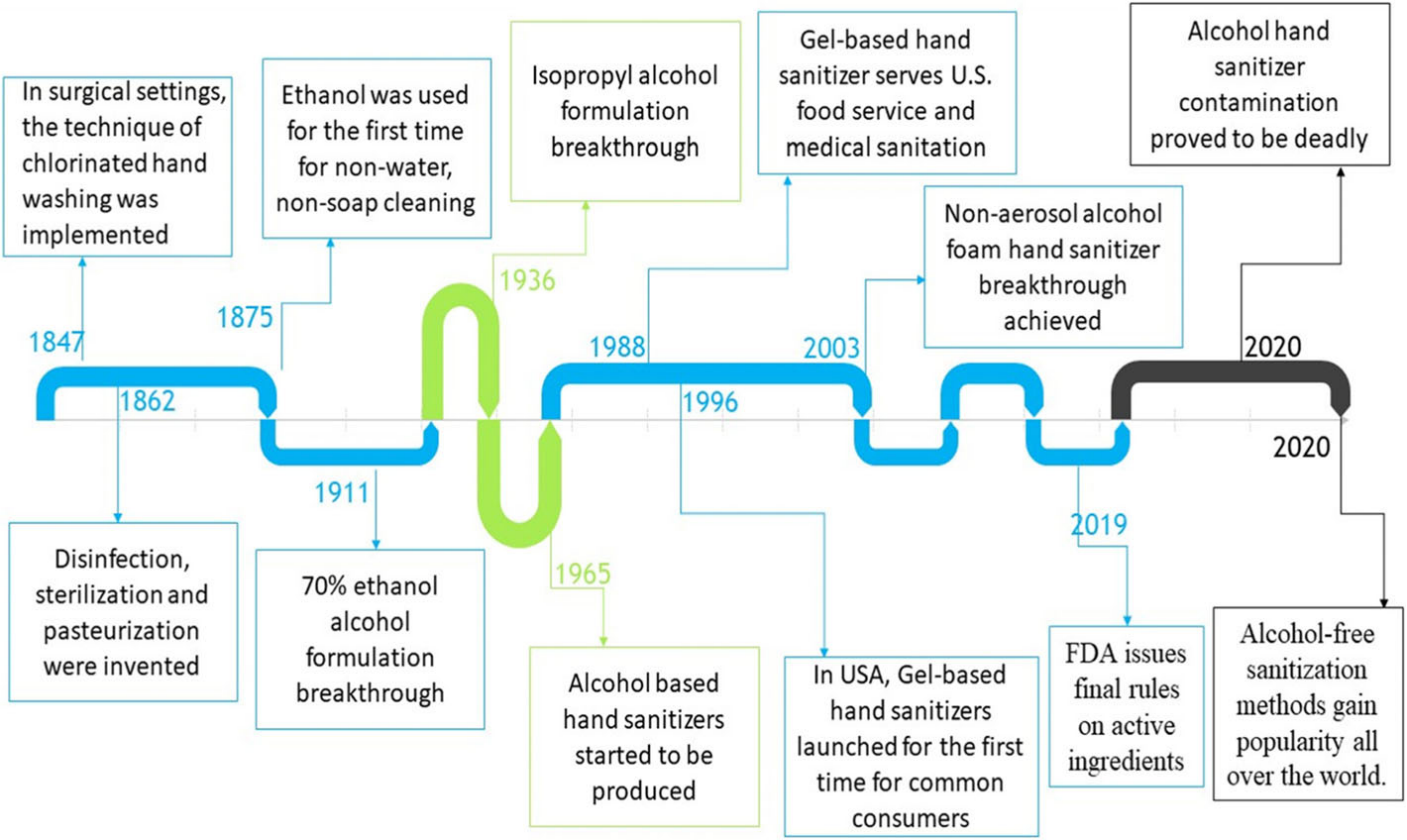
Timeline of hand sanitizer’s history [9, 10]

The timeline of hand sanitizer’s history [9, 10] Fig. 1.

**Table 1:** Recommended composition for preparing alcohol-based hand sanitizer by United States Pharmacopoeia Committee of Experts (USP) and World Health Organization (WHO) during the COVID-19 crisis [30, 31]

Components	Formulation 1: Ethanol antiseptic- 80% topical solution	Formulation 2: Isopropyl alcohol antiseptic- 75% topical solution	Formulation 3: Isopropyl alcohol antiseptic- 75% topical solution
Ethanol – 96%	833.3 ml	–	–
Isopropyl Alcohol- 99%	–	757.6 ml	–
Isopropyl Alcohol- 91%	–	–	824.2 ml
Hydrogen Peroxide- 3%	41.7 ml	41.7 ml	41.7 ml
Glycerol- 98%	14.5 ml	14.5 ml	14.5 ml
Water	1000 ml	1000 ml	1000 ml

## Relation between COVID-19 and hand sanitizer

### Mechanism of action of hand sanitizers against viruses

Viruses are structural infectious agents that contain genetic material like DNA or RNA. Viruses are encapsulated and protected by a protein envelope known as capsid. Viruses are further categorized as “enveloped” or “unenveloped.” Apart from the structural elements, it also contains a host cell which helps in propagation of viruses [11]. A common compound in virucides is N-Propanol. It is believed that it damages the membrane of the virus and it affects its decoupling and protein synthesis. For effective virucidal efficacy concentrations of not less than 60% and not more than 90% is enough. Alcohol that contains not more than 1% water is less virucidal than alcohol in the ranges mentioned above. Therefore, water is essential in the process of protein denaturation. Regardless of what processes alcohol affects, if not many, it eventually results in primary metabolic pathways, damage to the cell membrane, and loss of cell integrity. Alcohol-based hand sanitizers target the cover that protects the components of the virus. All the components are very critical for the virus. Therefore, it is assumed that after one of the components is targeted it may lose its ability to transfer from one host to another. This mechanism is compared to bacteria; it is observed that ethanol’s virucidal effect when compared to propanol is more effective against viruses of clinical relevance. It is also worth noting that in cases where only ethanol is not as effective against the viruses, adding acids can improve ethanol’s performance [12].

The SARS-CoV-2 virus was named because its genome sequence is similar to that of the SARS coronavirus (SARS-CoV) [13, 14]. CoV is related to the same gene beta coronavirus and has the same morphology as the single-stranded RNA virus [15, 16]. Ethyl alcohol at a concentration of 60 to 80% is a potent agent that inactivates all lipophilic viruses (such as influenza virus, herpes, and vaccinia and many hydrophilic viruses (e.g., polio) [17].

The WHO in 2015 recommended 80% concentration of ethanol and 75% concentration of isopropyl alcohol for “disinfectant: alcohol-based hand sanitizer” [18]. Ethanol seems to be more effective against bacteria than isopropanol and N-propanol. WHO also recommended that alcohol-based disinfectants showed results against emerging viruses too for example Zika, Ebola, and MERS [19, 20]. Another study showed that a little over 42.5% concentration of ethanol was able to destroy coronavirus in about thirty seconds [21].

### Efficacy of hand sanitizers on viruses

It is harder to study viruses than bacteria. Many studies have tried to test the efficacy of hand sanitizers on viruses. Use of alcohol-based hand sanitizers are recommended by WHO for protection against multiple viruses including the coronavirus as it has proven effective in quantitative suspension testing [19]. Other sterile sources containing isopropyl alcohol as the main ingredient also have proven effective against multiple enveloped viruses [17].

Studies have been conducted in which viruses have been externally applied or put-on fingers and hand sanitizers have successfully reduced the viral particles [22, 23]. Ethanol have been shown to be highly effectual against germs and most hospital-related viruses [24, 25]. Seventy to 80% alcohol concentration was enough for reliable inactivation in multiple viruses. Adequate activities can be attained with prolonged contact with the concentration of alcohol over time and prolonged contact with undiscovered virus.

A review of the literature on the effectiveness of handwashing against severe acute respiratory syndrome (SARS) transmission found that nine out of 10 small case-control studies showed that hand washing reduced the risk of social contamination [26]. Vivo evidence of viral inactivity after the use of hand sanitizers is not available by standard methods. Vitro studies have established that alcohol-based disinfectants can be effectual in reducing viral load [8].

The SARS-CoV-2 transmission has an incubation time of 10 days, which makes it easy to propagate through drops, contaminated hands or surfaces. Therefore, the effect of viral inactivity on all broadcasts should be considered [27]. Alcoholic disinfectants have been able to deactivate SARS-CoV-2 and MERS-CoV (also pre-activated coronaviruses) on living surfaces like plastic, glass, and metal [28]. A key limitation in analyzing the actual performance of hand disinfection is the recurring process of self-reported data gathering, which may not be the same and objective in terms of the frequency and method of hand washing [26].

## Hand sanitizer types [29]

**Fig. 2:**
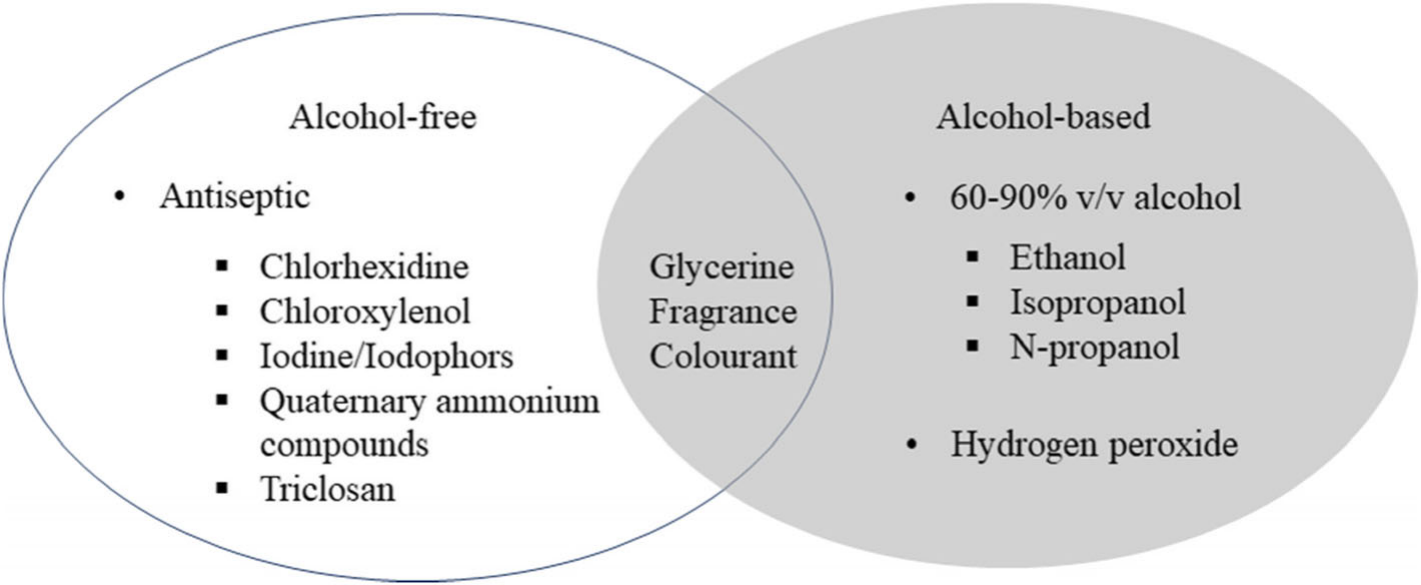
Hand sanitizer types (alcoholic compounds, non-alcoholic compounds, and commonly used excipients)

Hand sanitizers can usually be divided into two types: alcoholic or non-alcoholic. Alcohol-based hand sanitizer (ABHS) can contain alcohol, additives, and humectants to inhibit the growth and to kill the germs Fig. 2.

## Composition of hand sanitizers (Table 1) [30, 31]

## Different preparation methods for alcohol-based hand sanitizer gels [32]

### Direct addition method

Direct addition method as the name suggests is a method where in all the ingredients are added and mixed simultaneously but before the thickening agent. After the ingredients are mixed in the required compositions, the thickening agent is added. After the thickening agents are added, there are cases where there is a requirement of adding a neutralizing agent to maintain the pH level of the sanitizer to make it pH neutral. Modifications in the process is dependent on specific chemical compositions of ingredients in cases were the thickening agents not compatible with neutralizing agents.

### Inverse addition method

In this procedure, the thickener is pre-wetted usually with glycerol. Once this is done, the sanitizer ingredients are added gradually and mixed thoroughly. The name itself suggests that this is opposite to the above method. The addition and requirements of neutralizing agents is similar to the above method. This method is suitable for small scale productions of sanitizers.

### Other methods

High-viscosity solutions have the tendency to form lumps. We can use “hot/cold” technique to avoid this problem. This method is not suitable for components containing ethanol. Ethanol and other volatile components are added after a gel is prepared via hot cold technique during the cool down phase of the technique.

To avoid disadvantages of the above methods, like caking, a combination of the above methods can also be used for preparation of sanitizers. This means after preparation of a wet slurry, other ingredients are added using mechanisms used in addition method.

## Various dosage forms of hand sanitizer

The United States Centres for Disease Control and Prevention (CDC) promoted washing hands and use of disinfectants [33]. Hand sanitizers are commercially available in a variety of forms, such as alcohol or water-based hand sanitizers, which are commonly utilised in a hospital setting. Various types of distribution structure have also been developed, such as gel, spray, wipe, cream, or foam. Alcoholic hand sanitizer is recommended by the World Health Organization (WHO) on the basis of the proven benefits of its rapid work and the proven spectrum of bacterial activity that protects against germs [34–38].

## Marketed hand sanitizers

**Fig. 3:**
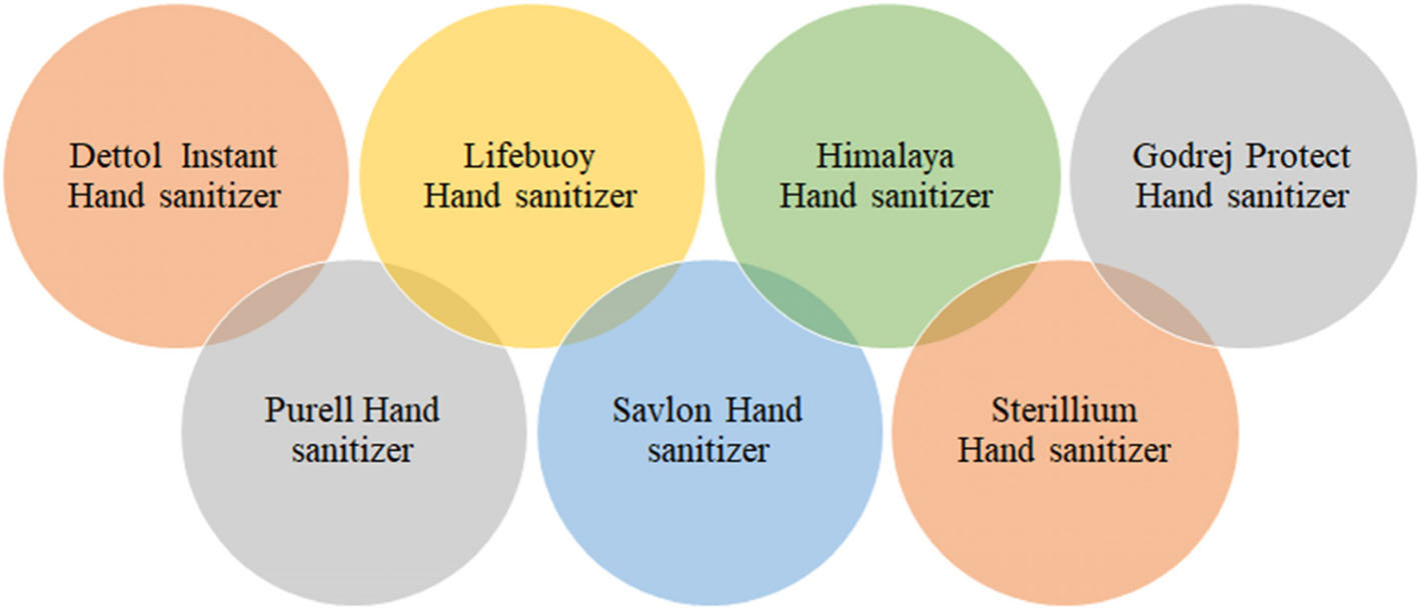
Well-known marketed hand sanitizers

There are several hand sanitizers of well-known brands that are available in the market as shown in Fig. 3.

## COVID-19 and the global hand sanitizer market

As COVID-19 has been exponentially spreading, the demand for hand sanitizers has also increased with the pharma companies and others producing hand sanitizers in huge quantities. People started buying them as a personal prevention tactic against the virus. This has suddenly led to a huge demand for raw materials for hand sanitizers. Raw materials like isopropanol were sold at higher prices as there was not enough stock due to sudden increase in demand. The demand has been met by innovations by the producers in the hand sanitizer industry and hand sanitizers have been produced in record numbers [39]. The hand sanitizer market has grown both in terms of revenue and sales in 2020 [40].

## Innovation in production of hand sanitizer during outbreak of SARS-COV-2

### Production of alcohol-based sanitizer from sweet potato residue in the COVID-19 emergency [41]

As coronavirus cases increased worldwide, hand sanitizer demand increased. So, to fulfil this demand, development of other new methods of producing alcohol-based hand sanitizer was the only way. Conversion of raw material from agriculture and food residue gives valuable bioproducts.

Sweet potato (Ipomoea batatas) is a source for manufacturing of bioethanol, as it has high amount of starch. This innovation technique was used in Brazil as Brazil is the 16th highest producer of sweet potato in the world and it produces 350,000 tons of sweet potato waste. Here, they develop the method of ethanol production from sweet potato waste. Greenhouse emissions also get reduced as the production of bioethanol from biowaste. Also, it increases new business opportunities in the food and agriculture field. Production of alcohol-based hand sanitizer from sweet potato residue was achieved as it showed favorable results and produced 1342 L of hand sanitizer per day.

### Benzalkonium chloride (BZK) for hand disinfection during COVID-19 [42]

Benzalkonium chloride hand cleaner, manufactured in an FDA-registered facility to cGMP requirements is readily available. The CDC’s inclusion of benzalkonium chloride for hand sanitizer in its current COVID-19 hand hygiene recommendation, which is clearly supported by the available literature would immediately ease some of the extreme pressures already strained on ABHR’s (alcohol-based hand rub) supply chains. Products containing benzalkonium chloride that comply with the current FDA OTC monograph may offer another option for hand disinfection. Extending the recommendations would also allow the immediate implementation of a viable alternative to hand washing in situations where the use of alcohol-based products is restricted due to concerns about alcohol abuse and the potential arming of ABHR (alcohol-based hand rub) products [42].

Benzalkonium chloride hand sanitizer was compared with 70% ethanolic hand sanitizer for a week and the results show that benzalkonium chloride hand sanitizer was more effective to decrease Staphylococcus aureus on the fingertips of healthcare workers [43].

### Production of ethanol-based hand sanitizer in breweries during COVID-19 emergency [44]

Due to increase in corona cases, disruption occurs in management of supply chain around the world. So, to solve this problem, many distilleries and breweries come to one conclusion that their facilities could help to produce hand sanitizer which follows the World Health Organization (WHO)-recommended formula and by considering all precautionary parameters they produced hand sanitizer safely and efficiently.

## Alcohol and soaps

Keeping hands clean so as not to get sick while reducing the spread of germs to others is an important and necessary step. The CDC has also emphasized that keeping hands clean can prevent a lot of diseases from spreading by removing dirt and microbes from the surface of the skin [45]. Both soap and alcohol disinfectant work on microbial lipid membranes by dissolving and thus disabling them. If water is not available, then a very good alternative is alcoholic disinfectants, and the alcoholic content should be nearly about 60% in the disinfectant.

Compared to soap, alcohol-based disinfectants do not kill all types of bacteria, such as noroviruses and clostridium difficile, which can cause common diseases [46, 47]. One major downside of sanitizers is that the liquid can evaporate before wiping evenly on all hands, thereby reducing the effect of disinfectants [48, 49]. Moreover, disinfectants are also ineffective when hands are covered by some chemicals or have dirt on them [50].

## Hand sanitizer versus soap

CDC has recommended washing hands with soap and water. It has multiple benefits like removal of pathogens and unwanted chemicals from hands. A 2016 systematic review showed that washing hands with soap is more effective than sanitizers to remove dirt and microorganisms [51]. However, when the effectiveness of soaps and disinfectants on bacterial inactivation were compared to different ethanol-based hand sanitizers, the vitro quantitative suspension test showed a 4 log 10% reduction (> 99.99%) in the tested envelope virus [52]. There are not enough studies on direct comparisons between soap and disinfectants. However, in some particular viruses and bacteria, soap and water is effective in comparisons with alcoholic disinfectants [19, 53].

In addition, washing the hands removes the skin’s own fatty acids that can lead to stressful skin, ultimately representing a potential gateway for germs [46, 54]. To overcome the limit of simple hand washing, hand sanitizers have been introduced that are effective in combating pathogenic microorganisms and also improve the condition of the skin by adding emollients [55, 56].

## Harmful effects of hand sanitizers on human health

### Ethanol poisoning

Ethanol is widely used as a disinfectant and alcoholic beverage. The possibility of skin absorption and skin cancer through carcinogenicity remains unclear due to the lack of current research [57]. There is no specific measure to assess toxic levels of ethanol disinfectants. Various studies have proven that acute exposures are not toxic. However, blood ethanol levels are affected with long-term exposures to ethanol-based hand sanitizers. In humans with 33% damaged skin, 70% ethanol is absorbed through the skin [58]. Moreover, exposure to ethanol-sensitive skin can cause systemic toxicity and reaction to the system. In cosmetics, it is also not suggested to use ethanol on injured skin. Eye irritation, skin dryness, cracking, redness, itching, and contact dermatitis can be caused by regular exposure to ethanol [59]. Studies have showed that ethanol sanitizers affect the concentration of ethyl glucuronide in urine. Acute alcohol poisoning can be caused by any daily home use items like alcoholic hand sanitizers (ABHS), mouthwash, cosmetics, and so on. Clinical symptoms appear at a certain concentration of alcohol in the blood. Lethal dose of ethanol can be life threatening. Symptoms begin 1 to 2 h after consuming ethanol-based hand sanitizers. Symptoms like vomiting, epigastric pain, and various depressions of the central nervous system are commonly seen. Ethanol poisoning has also been linked to hyperthermia, possible heart attacks, arrythmia, hypoglycaemia, ketoacidosis, and hypotension [60].

### Isopropyl alcohol poisoning

Higher molecular weight of isopropyl alcohol tends to be more lethal than ethanol poisoning. Such poisoning is commonly seen due to accidental ingestions. Studies and data have shown that the lethal dose was about 250 ml [61]. Exposure to the minimum dose was not a serious health problem and consuming 50% concentration of more than 25 ml isopropanol caused minor symptoms. Isopropyl alcohol has major clinical effect on children. People can be at a risk of long-term depression, hypotension, and central nervous system dysfunction after prolonged exposure to isopropyl alcohol. Isopropyl alcohol also irritates the mucous membranes in the stomach and may result in gastritis associated with ketosis, hypoglycaemia, respiratory depression, and high serum creatinine. A high dose can weaken heart muscle and its long-term use is conducive to rhabdomyolysis, myoglobinuria, and acute renal failure. Seventy percent of the deaths were associated with ingestion of ≥ 400 mg/dL in concentrations of 70% isopropyl solution [62, 63]. Isopropanol absorption through the skin can cause skin irritation, and prolonged and frequent exposure can cause blemishes, wrinkles, redness, and dryness [64].

### Toxicity of hydrogen peroxide

Hydrogen peroxide is only risky when consumed in high concentration. In some cases, it causes portal vein obstruction, abnormalities in the stomach, slight irritability, and vomiting of sous vide [65]. It creates toxic gases which when it comes in contact with tissues, it breaks down in oxygen and water. The presence of oxygen and water can cause air embolism in many organs [66].

### Child risk factor

Hand sanitizers come in colourful packaging with tempting flavors and children may lick it. Small doses are not harmful; however, young children are more likely to be poisoned by alcohol than adolescents [67]. Young children are more prone to liver issues when intoxicated with alcohol. Recent reports have shown that young children, including those with apnoea, acidosis, and coma, have been drinking alcohol-based hand sanitizers. A CDC research paper analysed data reported to the National Poison Data System (NPDS) on sanitizer exposures in the hands of children under 12–14 years of age [68]. In the first half of 2020, the U.S. reported that 9504 children under the age of 12 came in contact with a disinfectant and concluded that even a small amount of alcohol could harm children [69].

### Skin effects due to exposure to hand sanitizers

Studies have shown that immoderate use of disinfectants to prevent against the coronavirus has resulted in damaging the skin which has resulted in reducing its capabilities to fight against another virus. The use of skin disinfectants deprives the skin from sebum and water hence causing skin dryness. Dry and damaged skin is the focal point of many bacterial diseases and increases the risk of germs on the skin. Increased use of hand sanitizers has shown in surveys and studies that it increases the chances of getting norovirus [54, 70].

Hand sanitizers containing an alcohol can also dissolve the lipid levels of the skin and its lipid-dissolving effect is incompatible with the concentration of alcohol. Therefore, damaging lipid barriers and eventually it causes hand eczema [71, 72].

Once the barrier is broken, it eventually causes dermatitis. Symptoms like dryness, acne, wrinkles, burning, swelling, erythema and cracking are very common for dermatitis [73]. Currently, a study done on health workers with COVID-19 in China reported skin damage to the skin due to hand hygiene. Workers who cleaned up hands frequently have reported skin damages [74].

Eczema can be clearly distinguished from the place of disinfection with swelling, wrinkles. The part of hand where eczema has been detected may also turn into ulcer when it is exposed to disinfectant. In severe cases, the wounds may be swollen, and even damaged, with secondary infections [56, 71].

## Conclusion

Given the exploding nature of the pandemic, the world health machinery suggested regular use of hand sanitizers as one of the protection measures against the coronavirus. Hand sanitizers helps fight against the virus by damaging the cell membrane and therefore damages the components of the virus making it futile. Hand sanitizers have been highly effective to inactivate multiple viruses. Studies showed that alcohol-based disinfectants were effective in deactivating SARS-CoV-2 and MERS-CoV. Alcohol-based hand sanitizers includes components like ethanol, isopropyl alcohol, glycerine, and water and are available in various forms. There are multiple methods of industrial preparation of hand sanitizers. Industry experts invented various innovative methods to meet the requirement of the hand sanitizers. However effective the hand sanitizers are, they come with various adversarial effects. Ethanol exposure causes skin irritation, eye irritation, cracking of the skin, and redness and can also cause contact dermatitis. Isopropyl poisoning is also a threat due to long term use of hand sanitizers. Hydrogen peroxide is an important content of hand sanitizers, and it has proven to be highly risky when consumed in high concentrations. Children are also at a high risk of getting affected by hand sanitizers. Drinking hand sanitizers can cause serious harm to children. Skin is the most exposed to hand sanitizers. Extensive use of hand sanitizers can likely lead to hand dermatitis and hand eczema. An alternative to hand sanitizers as suggested by experts is hand washing with soaps. Though its limitations of not being available at all places, it has been found that soaps are highly effective to fight the virus and, in some cases, even more effective than sanitizers. Therefore, to combat the current pandemic hand sanitizers are a very effective way to keep the virus from spreading and affect us but it is also seen that extensive use of hand sanitizers can be harmful.

## Abbreviations

WHO: World Health Organization
DNA: Deoxyribonucleic acid
RNA: Ribonucleic acid
CDC: Centers for Disease Control and Prevention
ABHS: Alcohol-based hand sanitizer
USP: United States Pharmacopeia
FDA: Food and Drug Administration
ABHR: Alcohol-based hand rub
